# The Family Startup Program: study protocol for a randomized controlled trial of a universal group-based parenting support program

**DOI:** 10.1186/s12889-015-1732-3

**Published:** 2015-04-21

**Authors:** Tea Trillingsgaard, Rikke Damkjær Maimburg, Marianne Simonsen

**Affiliations:** Department of Psychology and Behavioural Sciences, Aarhus University, Bartholins Allé 9, 8000 Aarhus C, Denmark; Department of Clinical Medicine & Department of Obstetrics and Gynaecology, Aarhus University Hospital, Palle Juul-Jensens Boulevard 99, Skejby, 8200 Aarhus N, Denmark; Department of Economics and Business, Aarhus University, Fuglesangs Allé 4, 8210 Aarhus V, Denmark

**Keywords:** Parenting education, Primary prevention, Father-child relations, Early intervention, Child development, Perinatal care, Postnatal care, Community health services, Child abuse

## Abstract

**Background:**

Inadequate parenting is an important public health problem with possible severe and long-term consequences related to child development. We have solid theoretical and political arguments in favor of efforts enhancing the quality of the early family environment in the population at large. However, little is known about effect of universal approaches to parenting support during the transition to parenthood. This protocol describes an experimental evaluation of group based parenting support, the Family Startup Program (FSP), currently implemented large scale in Denmark.

**Methods/design:**

Participants will be approximately 2500 pregnant women and partners. Inclusion criteria are parental age above 18 and the mother expecting first child. Families are recruited when attending routine pregnancy scans provided as a part of the publicly available prenatal care program at Aarhus University Hospital, Skejby. Families are randomized within four geographically defined strata to one of two conditions a) participation in FSP or b) Treatment As Usual (TAU). FSP aims to prepare new families for their roles as parents and enhance parental access to informal sources of support, i.e. social network and community resources. The program consists of twelve group sessions, with nine families in each group, continuing from pregnancy until the child is 15 months old. TAU is the publicly available pre- and postnatal care available to families in both conditions. Analyses will employ survey data, administrative data from health visitors, and administrative register based data from Statistics Denmark. All data sources will be linked via the unique Danish Civil Registration Register (CPR) identifier. Data will be obtained at four time points, during pregnancy, when the child is nine months, 18 months and seven years. The primary study outcome is measured by the Parenting Sense of Competence scale (PSOC) J Clin Child Psychol 18:167-75, 1989. Other outcomes include parenting and couple relationship quality, utility of primary sector service and child physical health, socio-emotional and cognitive development.

**Discussion:**

The protocol describes an ambitious experimental evaluation of a universal group-based parenting support program; an evaluation that has not yet been made either in Denmark or internationally.

**Trial registration:**

ClinicalTrials.gov ID: NCT02294968. Registered November 14 2014.

## Background

### Specific background and explanation of rationale

Inadequate parenting is a significant public health problem and may potentially have life-long consequences for the affected children’s physical and mental health, educational attainment, labor market success, and family formation (see for example [1,2]). Inadequate parenting spans from childrearing practices characterized by lack of resources, knowledge, skills and confidence to severe forms of physical and emotional ill-treatment, sexual abuse, neglect, and exploitation. Most recent Danish estimates range from one in five families being at risk of inadequate parenting resources [3] to 0.05 per thousand children being at risk of terminal child maltreatment [4].

There are solid arguments for strengthening parenting support as early as possible. As a result of their dependency, vulnerability and relative social invisibility, infants are more exposed to risks related to inadequate parenting than older children. Infant cases are also the least likely to come to the attention of anyone outside the family that can monitor their care and safety [1]. Furthermore, research into early brain development indicates that the brain’s development can be physiologically altered by severe stress imposed by inadequate parenting during a child’s early years [5]. Insecure attachment and poor stimulation can reduce cognitive and emotional functioning and slow physical growth can increase the risk of illness in adulthood [6].

There are also sound economic arguments for early investments. Recently, Heckman and co-authors have emphasized the importance of investing early in particularly vulnerable children. Cunha and Heckman, for example, show theoretically that early investments not only have a large potential pay-off, they are also efficient in the sense that there does not exist an equity-efficiency trade-off, which is the case for later investments [7]. The reasons are that, in their model, skills acquired in one period persist into future periods and that skills produced at one stage raise the productivity of investment at subsequent stages. Importantly, skills are multidimensional and are likely to complement each other.

The transition to parenthood provides optimal opportunities for reaching families. Couples expecting or having their first child are often eager to prepare for their roles as parents [8] and in Denmark, high rates of attendance are observed in preventive or educational programs offered to all couples during transition to parenthood [9].

To date, some prevention research has shown promise in enhancing parenting practices during the transition to parenthood, but most of these studies have focused on high-intensity service delivery (e.g. home visits) to specific subsets of abusive parents or disadvantaged families (e.g. [10-12]). This paucity of evidence regarding large scale primary prevention programs raises concerns that scarce resources may be wasted through investment in well-intentioned initiatives whose effectiveness may never be proven.

### Effects of universal parenting programs during the transition to parenthood

Universal approaches to parenting support have as their general aim to enhance the quality of the early family environment in the population at large (as opposed to targeting a specific problem in identified cases of families and children with special needs). For example, in a randomized trial of prenatal parent education, all nulliparous women allocated to routine care were included, and effects related to pregnancy worry, the birth process, and prenatal confidence in breastfeeding were found [13-15]. To the authors’ knowledge, no experimental design study was previously conducted on large scale implementation of universal group based parenting support with both pre- and postnatal program elements. The existing experimental studies are small scale or university-based and deliver infant care education primarily to mothers in group or class formats (for reviews see [8,16]). Data from these studies provide for some optimism with regard to the ability of infant care education to produce small to moderate effect size improvements on maternal adjustment and postpartum parenting skills [8,17]. However, the literature is sparse when it comes to how the effects from university settings generalize, when programs are implemented at the population level and what the long term effects and consequences for child outcome may be. Also, studies including fathers are few and with mixed conclusions. This may be particular surprising given the amount of research that point toward a direct link between fathers’ early involvement and the child’s social and cognitive development (for review see [16]).

When implementing the “Building Strong Families” parenting program in the US, fathers and family stability were the primary target, yet no positive effects were found on relationship satisfaction, marriage or father involvement [18,19]. In line with this, Trillingsgaard, Baucom, Heyman & Elklit found no effects of the couple-focused intervention “PREP” when offered to Danish men and women during pregnancy [20]. Halford, Petch, & Creedy compared effects of a) a maternal education program and b) a couple-focused education program “Couple Care”, and found effects on mother reports only [21]. Among more successful programs, Doherty, Erickson, & LaRossa examined an 8-session group based and couple-focused intervention and found positive effects on fathers’ skills in interacting with their babies and their involvement with child on work days but not home days [22]. Also, a study conducted by Schulz, Cowan & Cowan found that 24 weekly group meetings involving fathers in discussions of parenthood and the couple relationship helped both partners sustain relationship adjustment for up to five years after the birth [23].

In summary, the existing literature suggests that universal group-based parenting support programs may have at least short term positive effects on parenting outcome, when programs are conducted in controlled settings and are mother-child focused. When it comes to extending the scope and aim of these types of interventions, we have mostly theoretical and political arguments. The effects to be gained from intervening at the population level, also targeting fathers, and examining longer term child outcome are largely unknown.

### Rational of the Family Startup Program

The FSP is a structured format for implementing pre- and postnatal parenting support groups that prepare new families for their roles as parents and enhance parents’ access to information sources of support, i.e. social network and professionals representing community resources (for manual, see [24]). The program content includes handling family finance, choice of paternity leave, couple communication, breastfeeding, network formation, ensuring dental health, sensitivity toward child signals, child rearing discipline, help-seeking behavior, home safety, and more. Thus the FSP has not one but a wide range of possible learning outcomes most of which relate to a central rational of empowering parents by enhancing their sense of competence and lowering parental stress across the transition to parenthood. A second formulated rational behind the program is that strengthening father involvement, social network formation and access to family services will serve to enhance family relationships, including parenting, coparenting and couple relationship quality [24]. Through participation in FSP families receive a long term connection with a health visitor in the community and are introduced to a broad range of community services. This can be expected to benefit optimal health service utility and ease access to resources for families and children with need of additional support. Improved child outcome is the ultimate goal. No scientific evaluation of program effects of the FSP on child or family outcome was previously conducted.

### Specific objectives and hypotheses

The aims of this study is to determine whether a group-based parent support program can lead to:

1. Early improved parental sense of competence (primary outcome)
2. Continued improvement in parental sense of competence (secondary outcomes)
3. Reduced parental stress (secondary outcomes)
4. Improved quality in early family relationships, that is improved quality of parenting, coparenting and couple relationship quality (secondary outcomes)
5. Improved efficiency in service delivery, that is increasing surveillance of families at risk, and enhancing families’ utility of community resources, social network and family services (tertiary outcomes)
6. Improved child outcomes including better physical health and improved socio-emotional and cognitive development (tertiary outcomes)
7. Heterogeneity of effects across family type with disadvantaged families and families met with a more mature program implementation gaining more from participation (tertiary outcomes).

## Methods

### Trial design

The study is an individually superior randomized controlled trial with two parallel arms. The experimental group is offered FSP and the control group TAU.

### Participants

#### Inclusion criteria

Parents, mothers as well as their partners, expecting their first child in the municipality of Aarhus are eligible for the study. Mothers are included if interested regardless of the fathers’ decline. Biological fathers, registered partners, as well as non-registered partners are eligible.

### Exclusion criteria

Parents are excluded from the study if they are under the age of 18 years, not capable of managing own legal affairs, are found by their general practitioner to be in need of drug or alcohol abuse treatment, if they choose not to accept any routine pregnancy scans, or were not willing to provide signed consent. These exclusion criteria are chosen to secure that study participants are legally responsible and that we are able to recruit them via the Department of Obstetrics and Gynaecology in connection with the standard pregnancy scans.

The father or partner is not included if the mother declines participation. The mother can choose to attend the FSP with a friend (i.e. a sister, friend or neighbor) but friends are not eligible for the study. Finally, individuals are excluded if they do not have sufficient Danish skills. The rationale for this is the group nature of the program.

### Setting

This study is conducted in the municipality of Aarhus, Central Region Denmark. The inclusion began on November 24, 2014. Parents will be recruited through Aarhus University Hospital, Skejby, Department of Obstetrics and Gynecology. All pregnant mothers who fulfill the inclusion criteria and are referred from the general practitioner to receive pregnancy care at the hospital will be informed about the study. Information about the study and recruitment will take place at the Ultrasound Department at Aarhus University Hospital in week 12 or 19 of gestational age.

### Interventions

During the project period, the experimental group will receive the FSP and the existing publicly available pre- and postnatal care services.

### The Family Startup Program

FSP is a group-based manualized program for implementing pre- and postnatal parenting support that prepares new families for their roles as parents. The approach is inspired by community work conducted in the town of Leksand, Sweden [25]. The Danish manual details the content for each session, both in terms of themes, in terms of time (down to five minute intervals), and in terms of teaching material. The manual is copy-righted but for purposes of replication can be required from the Department of Children and Youth in Aarhus Municipality (“Mødeguiden”; [24]).

The program has an explicit focus on the enhancement of parents’ social network. Parents assigned to the FSP are offered 12 group (ideally consisting of 9 families) meetings whereof 2 meetings take place before the birth of the child, starting around week 28 of the pregnancy, and 10 meetings are scheduled after the birth of the child. The program lasts until the child is around 15 months old. Meetings are held from 4.30-6.30 pm to minimize interference with families’ working lives.

### Meeting structure, didactics, and teaching material

The program is delivered in two-hour face-to-face sessions with a health visitor as the permanent group leader. Participants are introduced to a broad range of community resources by means of oral presentations from e.g. financial advisers, legal advisers, psychologists, physiotherapists, and family counselors providing them with information about the specific theme of the session. The timing of sessions and their content are shown in Table 1.

**Table 1:** **Timing of session and content in Family Startup**

**Meeting**	**When?**	**The topic of the day/presentations**	**Teacher(s)**
1	Week 28	Welcome and presentation of the program	Heath visitor
		Finances and changes in the budget of the family Networking	Financial adviser
		What does an infant need	Nurse
2	Week 32	How to ensure and insure each other?	Legal adviser
		Legal paternity- what does it mean?	Health visitor
THE BIRTH OF THE CHILD			
3	Child 1 month	Relationships, communication, and sleep	Marte Meo therapist
		The everyday life with an infant	Health visitor
4	Child 2 months	Motor development in infants	Physiotherapist
		The infant’s basic needs	Health visitors
		Psychological reaction after the birth	Health visitor
5	Child 3 months	Family dynamics and interaction	Psychologist and health visitor
		Help and support- what are the possibilities?	
6	Child 4 months	Food and meals	Health visitor
		Medical check-up of the child	
7	Child 5 months	Preventing accidents	
		When your child is sick	Health visitor
8	Child 6 months	Language development	Language counsellor
		Child development	Health visitor
9	Child 7 months	The labor market and daycare enrolment	Pedagogue
		Sleep during day and night- how to get better sleep	Health visitor
10	Child 8 months	The personality of your child and independence	Health visitor
		The child’s first tooth	Dentist
11	Child 10 months	Family dynamics	Family counselor/pedagogue
		The everyday life with your child	Health visitor
12	Child 15 months	Upbringing: the everyday life and challenges in the family Resilience	Health visitor
		Goodbye	

The group leader facilitates discussion and arranges exercises before and after the oral presentations. The fixed structure of the meetings is as follows:

1. Follow-up on last meeting
2. Presentation of first theme of the day
3. Discussion and debate based on presentation
4. Summary of discussion
5. Break and refreshments/dinner
6. Presentation of second theme of the day
7. Discussion and debate based on presentation
8. Closing words, next meeting’s themes, evaluation.

The teaching style is dialogic and participants are directly involved through discussion, both in plenary sessions and in smaller groups (mixed gender, single gender, couples on their own). The manual includes PowerPoint presentations, video material, exercise sheets, evaluation sheets, and teaching instructions.

### Adherence

A set of procedures will ensure that the program is delivered to meet the standardized version of FSP. As described, program fidelity is supported by the detailed manual (“Mødeguiden”, Guideline for meetings in Danish; [24]). All health visitors, who will be group leaders, participated in a 3-day workshop on facilitating family group meetings prior to the study. A program manager (health visitor) is employed full time and will visit groups during sessions to facilitate and ensure quality of delivery. A brief measure of satisfaction with program will be obtained from all parents at three random sessions during the course of the program. Further, the group leader fills out a checklist at the end of each session, monitoring whether or not the main content was covered.

### Treatment as usual

The existing publicly available pre- and postnatal care service includes two group-based sessions of birth preparation, routine pregnancy visits to the general practitioner and midwife, and postnatal home visits by nurse. Specific pre- and postnatal care services target families with a certain profile (history of loss, history of depression, parents expecting twins or young mothers). None of the existing services will be affected by the addition of the FSP and Randomization. *All* families will be offered these existing services, regardless of their treatment assignment.

### Measures including outcomes

Our analyses will employ three main data sources with information about parents and children: survey type information, administrative data from health visitors, and administrative register based data maintained by Statistics Denmark. All data sources will be linked through the unique Danish Civil Registration Register (CPR) number and data will only be accessed in anonymous form.

Survey data will be collected by an email sent to both partners in the family with personal login to a web-based survey using the online survey solution *SurveyXact*. Two reminders are sent by email. Participants can choose to answer questionnaires through the telephone call if preferred. Survey data will be obtained at four time points, during pregnancy, when the child is nine months, 18 months and seven years. No monetary incentives are used to motivate participation. Lottery prizes with a value of maximum DKK 3,000 per prize will be drawn from the pool of project participants, who fill in the questionnaire.

### Background information and control variables

Information about parent age, gender, marital status and relationship stability, educational background, employment status, previous psychiatric/therapeutic treatment, smoking, place of birth, siblings, and receipt of welfare will be collected through survey single items during pregnancy or obtained through registers. Financial strain is measure with a 3-item scale [26], and social desirability is measured with 10 items adapted from the 31 items version of the Social Desirability Scale [27].

Background information on eligible individuals who do not enrol in the study is available from register-based data. In addition to these data we will ask decliners about their language skills, educational level and reason for decline.

### Primary study outcome

The FSP has a range of possible learning outcomes most of which are linked with the overall explicit goal of empowering parents. To tap into the parents’ own experience of parental capability and self-esteem we use as our primary study outcome the Parenting Sense of Competence scale (PSOC) measured at nine months. The PSOC measures parental self-esteem on two dimensions: Satisfaction and Efficacy. Satisfaction section examines the parents’ anxiety, motivation and frustration, while the Efficacy section looks at the parents’ competence capability levels and problem-solving abilities in their parental role. The constructs of satisfaction and efficacy are closely linked with a host of positive family interactions as well as with positive child development [28]. The total score of PSOC is calculated as the sum of 16 items, and has a possible range of 16 to 96. The PSOC was originally developed by Gibaud-Wallston and Wandersman to measure parents’ perceived competence with their infants [29]. Johnston and Mash changed item wordings from “infant” to “child” and validated the measure for use with parents to elementary school-aged children [30]. As the current study will obtain parental reports from both parents across seven years, it is of importance that the scale has previously demonstrated utility across child age, child gender and parent gender [28]. Furthermore, the PSOC appears to be sensitive to changes resulting from brief parenting support and this was also found in a non-clinical sample of Scandinavian parents [31]. A Danish translation and back-translation of the scale was conducted prior to the current study (Lange AM, Frantzen KK: At være forælder. Unpublished).

### Secondary study outcomes

*PSOC* measured at 18 months and seven years.

*Parenting stress* is measured with the Parenting Stress Scale [32] at nine months, 18 months and seven years.

*Parenting Quality* is measured at nine and 18 months with the Parenting Scale [33] and we intend to use the HOME inventory as nurse administrative data [34] at nine months. At nine and 18 months, a measure of father-involvement developed for the study and a single item on breastfeeding duration will be included.

*Quality of the Couple Relationship* is measured with the Couple Satisfaction Index [35] during pregnancy at nine months, at 18 months and at six years, and the Coparenting Relationship Scale [36] at nine months and 18 month, and divorce rate at seven years.

### Tertiary study outcomes

*Utility of primary sector service* is measured with register-based information on visits to the general practitioner; administrative data on referrals from primary sector, and with a questionnaire on help-seeking behavior adapted from the Attitudes Toward Seeking Professional Psychological Help Measure [37].

*Child physical health, socio-emotional and cognitive development* is measured at nine months by nurse observations of height and weight and ratings online and gross motor skills, problem solving and social skills during home visit. Socio-emotional development is measured with the Ages and Stages Questionnaire [38] at nine months and 18 months. Information about timing of enrolment in and type of non-parental child care and information on school starting age is obtained from registers. These choices have been shown to have long-term effects on child outcomes [39-41].

*Satisfaction with service delivery* is measured with evaluation sheets collected from families receiving the FSP by health nurses at the end of the third program session. A randomization procedure determines which specific three sessions are evaluated within each group. The evaluation sheet covers satisfaction with group sessions, group atmosphere and continuity and use of group network. Information on attendance to sessions, dropout and reasons for dropout is collected by the health nurse program leader at each session.

### 2.E Sample size

Meta-analytic studies on the effects of parenting education with expectant and new parents found on average small effects on measures of parental adjustment (i.e. PSOC) (*d* = .21, [42]). Stronger effects emerged if interventions included more than five sessions, included an antenatal and postnatal component and were led by professionals rather than semiprofessionals. Thus, we expected *d* = .20 to be a conservative estimate of effect on the primary outcome in the present study.

Findings on our secondary outcomes are more varied depending on the measure used. Parental stress as measured by the Parenting Stress Scale showed effect sizes of *d* = .20 [16] and parenting quality as measured with the HOME inventory (nurse observation, [34]) produced average effect sizes of *d* = .35 SD units [16]. Studies relying on parental self-report found somewhat lower effects; [36], for example, found small effects on coparenting quality (*d* = .18 SD units) and somewhat better effects on parenting quality (ranging from *d* = .30 to *d* = .36), and moderate size effects on relationship satisfaction (*d* = .43). In this study we use both the HOME inventory as well as Feinberg’s measures of parenting and coparenting/relationship satisfaction. Thus we expected *d* = .20 to be a conservative estimate of effects on the secondary outcomes in the present study.

Our primary as well as secondary outcomes all produce interval data that are not cardinal in nature. Thus, the value of a given outcome has no meaning in itself. This is clearly in contrast to many outcomes studied in the medical sciences (e.g. number of days admitted to hospital after surgery). One way of anchoring expected effect sizes is to compare to effects of other interventions. In our case, an effect size of .20 in terms of for example parental stress is comparable to average effects of other types of parenting education [16].

Our power analyses use the 2013 version of the Optimal Design software developed by Spybrook and collaborators. Since treatment in this subproject is carried out at the group-level, we conservatively use the two-level cluster-randomized version with family-level outcomes and ignore the presence of covariates. Assuming that 20% of the variation lies between groups and using a power of 0.80 and significance level of 0.05, we are able to detect an effect size of 0.2 with around 230 groups (115 groups in each condition) or a total of 2,070 families.

Assuming that 25% of study participants drop out after randomization and prior to the nine months follow-up, we will need to recruit 280 groups or 2,520 families. According to Statistics Denmark, 4,000 children were born in Aarhus in 2012. Since about 45% of all Danish live births were first born children, and assuming that 80% choose to participate in the research project, detecting such an effect size will roughly require a 1½ year trial; see also 3.A below.

Groups are considered as “did not receive intervention” if 50% of the mothers in a FSP group drop out or if more than two group meetings are cancelled due to group disagreement.

We consider women or men as dropouts from the program if they miss two consecutive meetings without reasonable reason or apologies (such as illness). We categorize women or men as “did not receive intervention” if they do not attend at least two of the postnatal sessions.

We will not base the continuation of recruitment on interim analyses. This is primarily because we expect the intervention to be associated with very low risk for participants. There may be smaller inconveniences, at least for some, associated with the level of time consumption from participating in a program such as FSP. Nevertheless, we will conduct interim analyses and report preliminary results when sample size allows for power of 0.80 to detect an effect size of 0.2 between conditions at the family-level (*N* = 800 families), ignoring group level variation. As explained, however, participation is entirely voluntary and will not affect access to other family services provided by the municipality or the region. Even if the program does not significantly improve participants’ primary outcome in short-term preliminary analyses, this will not lead us to discontinue the program thereby ignoring possible secondary, tertiary and longer term outcomes.

We will perform a dropout analysis that characterizes dropouts in terms of pre-intervention background variables (see list above) if more than 1/3 of study participants (mothers) drop out after initial randomization and prior to the nine months follow-up.

We will discontinue the study if less than 40% of those approached in connection with standard pregnancy scans have accepted to participate in the study after the first six months of recruitment. Similarly, we will discontinue the study if after six months of recruitment more than 2/3 of the women assigned to the FSP condition have dropped out after their initial randomization.

### Randomization

Families will be randomized upon receipt of their signed consent form. Randomization is performed at the family level. In order to keep travelling distances relatively short, randomization is carried out within four strata defined by geographic district in the municipality. We computerize the randomization via an unpredictable random sequence using Java. Critically, this offers allocation concealment. In practice we generate a uniformly distributed random variable on the (0,1) interval. If the assigned value is larger than 0.5, study participants will be offered FSP. If the assigned value is 0.5 or lower, study participants will be offered TAU.

Our aim is to form a new FSP group once at least eight or maximum nine families are assigned to treatment, with the additional aim that the maximum spread in due dates is five weeks. If both aims cannot be fulfilled in practice, the maximum spread in due dates will be prioritized. Hence the group composition will be random. An email with a personal login to the baseline questionnaire is automatically sent upon receipt of the signed consent form. After two automatically generated email reminders (if questionnaires are not filled in) the respondents are notified of their allocation to condition. Respondents who do not fill in questionnaires remain in the study and data are obtained through registers.

In case our restriction on the maximum spread in expected date of confinement causes groups to consist of less than seven families, the group will continue in FSP. However, if this occurs more than four times in a given geographic district during the first three months of project inclusion, we will switch to a block randomization principle within that particular district: once between seven to nine families are assigned to the *study* (keeping the restriction on maximum spread in expected date of confinement) we will create a group and randomization will be carried out at the group level instead.

### Implementation

Randomization and allocation into FSP and TAU will in practice be carried out by use of the program SurveyXact/Results owned by Rambøll Survey; the company that also collects the survey data. Rambøll Survey will code the randomization mechanism, which is concealed to the research team.

### Blinding

The research team and families remain blind to study condition during recruitment, consent and baseline questionnaire, though blinding after this point in time is not possible.

### Statistical methods

Reporting of results will follow the guideline of the CONSORT- statement. Statistical analysis will be intention to treat. The level of significance will be 0.05. The primary endpoint is PSOC at the nine month follow-up. The intention-to-treat analysis will compare FSP with TAU using both simple two-sample t-tests, non-parametric rank based tests (Wilcoxon), and linear regressions (ANCOVA) that control for pre-randomization variables (parents’ age, marital status, educational background, employment status, previous psychiatric/therapeutic treatment, smoking, place of birth, own number of siblings, receipt of welfare, financial strain, and social desirability). This latter analysis will cluster errors at the FSP group level. Except for the divorce rate at the seven year follow-up, which is binary in nature, all statistical analyses of effects on secondary outcomes will follow our strategy for the primary outcome. For divorce rate, we will use simple two-sample t-tests as well as a logistic regression that control for pre-randomization variables. Similar principles will be applied to the analysis of tertiary outcomes.

In addition to intention-to-treat analysis, we will also perform contamination adjusted intention-to-treat analysis, which complements the intention-to-treat approach by producing a better estimate of the benefits and harms of receiving a treatment. This method uses the statistical technique of instrumental variable analysis to address contamination [41-46].

In order to test whether different subpopulations experience differential effects from FSP participation (tertiary outcomes), we will define subpopulations based on pre-randomization variables. The null hypothesis is that subgroups are affected equally by FSP participation. The alternative hypothesis is that subgroups experience differential effects. We will consider mothers with above high school education versus mothers with high school or below; mothers aged 23 or above versus mothers aged below 23; mothers who experienced previous psychiatric/therapeutic treatment versus mothers who did not; families who have experienced financial strain versus families who did not; and families with low relationship stability versus families with high stability. We will, in addition, consider subgroups recruited early (during first six months after study initiation), mid-period (during months 7–12), and late (after month 12). In practice, we will test for significance of subgroup-treatment interactions using t-tests and perform a joint F-test for significance of the entire set of interactions [47].

Because our study collects multiple outcomes, permutation testing methods, and a step-down procedure will be applied to account for the increased likelihood of false discoveries [48]. This is adopted in combination with a naïve evaluation strategy (which examines each outcome individually).

Exploratory analyses that in addition to pre-randomization controls include indicators for the identity of the responsible group leader will be performed to control for variation in program delivery across health visitors. Exploratory analysis will also investigate whether initial group composition affects family outcomes. We will define group composition in terms of the shares of the members of a FSP group that belong to the various subgroups detailed immediately above. Note that group composition by construction will be random conditional on due date and strata.

Finally, as described, we will perform a dropout analysis if more than 1/3 of study participants drop out after initial randomization and prior to the nine months follow-up. In practice we will run a logistic regression of a binary variable indicating dropout as the dependent variable with pre-randomization controls as the independent variables.

### Dates defining periods of recruitment and follow-up

Recruitment began on November 24, 2014. Inclusion will continue at least until May 1, 2016. As detailed above, survey data will be obtained at four time points, during pregnancy, when the child is 9 months, 18 months, and 7 years. We will follow the families continuously through register-based data until the child turns 7 years old.

### Harms

The program consists of a network component (interaction with other couples who are also expecting their first child) and an information component provided by health professionals and others such as financial advisors, child dentists, and family counselors. There may be smaller inconveniences, at least for some, associated with the level of time consume from participating in a program such as FSP. As explained above, however, participation is entirely voluntary and will not affect access to other family services provided by the municipality or the region. Most meetings are held after 3 pm and 10 out of 12 are held from 4.30-6.30 pm. This is to minimize interference with families’ working lives. For all these reasons, we expect the intervention to be associated with very low risk for participants.

### Registration numbers and name of trial registry

The project is registered with Clinicaltrials.gov; ClinicalTrials.gov ID NCT02294968.

The project has been approved by the Regional Ethical Committee (Central Denmark Region); registration number ESDH 1-10-72-109-14.

The project has been approved by Danish data protection agency; registration number 2014-41-3016.

### Protocol availability

This protocol will be made available at www.econ.au.dk/familieivaerksaetterne.

## Discussion

The protocol describes an ambitious experimental evaluation of a universal group-based parenting support program that is currently being rolled out large scale in Denmark. Such an evaluation has not previously been made either in Denmark or internationally.

## Abbreviations

CPR: Central Personal Register
FSP: Family Startup Program
PSOC: Parental Sense of Competence
TAU: Treatment As Usual
